# Molecular Diagnosis and Phenotypic Variability of Noonan Syndrome: Experience from a Romanian Multicenter Study

**DOI:** 10.3390/diagnostics16081207

**Published:** 2026-04-17

**Authors:** Florina Victoria Nazarie, Mihaela Amelia Dobrescu, Cecilia Lazea, Ana Adriana David, Crina Șufană, Simona Bucerzan, Simona Sorana Cainap, Raluca Rancea, Oana Stănoiu-Pînzariu, Ionela Maria Pascanu, Radu Anghel Popp, Laura Ancuta Pop, Călin Lazăr, Camelia Alkhzouz, Diana Miclea, Romana Vulturar

**Affiliations:** 1 Discipline of Medical Genetics, “Iuliu Hațieganu” University of Medicine and Pharmacy, 6, Pasteur St., 400349 Cluj-Napoca, Romania; nazarie.florina.victoria@elearn.umfcluj.ro (F.V.N.); anghel.popp@umfcluj.ro (R.A.P.); 2 Department of Medical Genetics, University of Medicine and Pharmacy of Craiova, 2, Petru Rareș St., 200349 Craiova, Romania; amelia.dobrescu@umfcv.ro; 3 Regional Centre of Medical Genetics Dolj, Emergency County Hospital Craiova, 200642 Craiova, Romania; 4 1st Pediatric Discipline, Mother and Child Department, “Iuliu Hațieganu” University of Medicine and Pharmacy, 68, Calea Moților St., 400370 Cluj-Napoca, Romania; sbucerzan@umfcluj.ro (S.B.); calin.lazar@umfcluj.ro (C.L.); calkhuzouz@umfcluj.ro (C.A.); diana.miclea@umfcluj.ro (D.M.); 5 1st Pediatrics Clinic, Children’s Emergency Clinical Hospital, 68, Calea Moților St., 400370 Cluj-Napoca, Romania; ana.adri.david@elearn.umfcluj.ro (A.A.D.); crina@sufana.ro (C.Ș.); 6 Medical Genetics Compartment, Children’s Emergency Clinical Hospital, 68, Calea Moților St., 400370 Cluj-Napoca, Romania; 7 2nd Pediatric Department, Clinical Children Hospital, 3-5, Crisan St., 400124 Cluj-Napoca, Romania; cainap.simona@gmail.com; 8 2nd Pediatric Discipline, Mother and Child Department, “Iuliu Hațieganu” University of Medicine and Pharmacy, 3-5, Crisan St., 400124 Cluj-Napoca, Romania; 9 Department of Cardiology, “Niculae Stancioiu” Heart Institute, 19-21, Motilor St., 400001 Cluj-Napoca, Romania; rancea.raluca@elearn.umfcluj.ro; 10 PhD Student Doctoral School, “Iuliu Haţieganu” University of Medicine and Pharmacy, 8, Victor Babes St., 400012 Cluj-Napoca, Romania; 11 Department of Endocrinology, “Iuliu Hațieganu” University of Medicine and Pharmacy, 3, Pasteur St., 400349 Cluj-Napoca, Romania; oana_pinzariu@yahoo.com; 12 Endocrinology Clinic, Cluj County Emergency Clinical Hospital, 3, Pasteur St., 400349 Cluj-Napoca, Romania; 13 Department of Endocrinology, George Emil Palade University of Medicine, Pharmacy, Sciences and Technology of Târgu Mureș, 46, Gheorghe Marinescu St., 540136 Târgu Mureș, Romania; ionela.pascanu@umfst.ro; 14 Genomics Department, MEDFUTURE-Institute for Biomedical Research, “Iuliu Hațieganu” University of Medicine and Pharmacy, 23, Gheorghe Marinescu St., 400337 Cluj-Napoca, Romania; laura.pop@umfcluj.ro; 15 Discipline of Cell and Molecular Biology, “Iuliu Hațieganu” University of Medicine and Pharmacy, 6, Pasteur St., 400349 Cluj-Napoca, Romania; romanavulturar@gmail.com; 16 Association for Innovation in Rare Inflammatory, Metabolic, Genetic Diseases INNOROG, 400497 Cluj-Napoca, Romania

**Keywords:** Noonan syndrome, RAS/MAPK pathway, RASopathies, *PTPN11*, *SOS1*, *LZTR1*, *RAF1*, pulmonary valve stenosis, short stature

## Abstract

**Background**: RASopathies represent a clinically and genetically diverse group of syndromes resulting from germline mutations in genes regulating the RAS/mitogen-activated protein kinase (MAPK) signaling cascade. **Methods**: The aim of this study was to describe the clinical features and genetic variants identified in patients with genetically confirmed Noonan syndrome (NS) in a limited cohort from Romania. A total of 25 patients with positive genetic testing for NS-associated genes were included. Genetic testing was performed primarily using next-generation sequencing. **Results**: A total of twenty-six variants were identified in twenty-five patients, as one patient carried two pathogenic variants in the *PTPN11* gene (c.188A>G and c.922A>G). Of these variants, twenty-four (92.31%) were classified as pathogenic and two (7.69%) as variants of uncertain significance (VUS). Pathogenic variants were found in different genes, including *PTPN11*, *LZTR1*, *SOS1*, and *RAF1*, with *PTPN11* being the most frequently affected gene. Males predominated (17/25), with a male-to-female ratio of approximately 2:1. Two patients inherited the pathogenic variant from an affected parent. Cardiovascular involvement was present in 21 patients (84%), with pulmonary valve stenosis (PVS) being the most common finding (48%), followed by hypertrophic cardiomyopathy (16%). Additional cardiac anomalies included atrial septal defect, valvular regurgitation, dysplastic valves, coarctation of the aorta, and sinotubular junction narrowing. Short stature was observed in 64% of patients, and craniofacial dysmorphism was present in 96%. Cutaneous, ectodermal, dental, ophthalmologic, and auditory manifestations were variably observed. **Conclusions**: Although based on a limited cohort from Romania, this study provides insights into clinical features suggestive of NS. Our findings highlight the genetic heterogeneity of NS and emphasize the importance of comprehensive genetic testing for confirming diagnosis, guiding clinical management, and supporting family counseling.

## 1. Introduction

RASopathies comprise a clinically and genetically heterogeneous group of disorders caused by germline mutations in genes encoding components of the RAS/mitogen-activated protein kinase pathway (MAPK). This pathway is central to cell cycle control, growth, differentiation, and apoptosis, and its dysregulation disrupts normal development. Most disease-causing variants are gain-of-function and increase pathway signaling. Multisystem involvement is common—particularly affecting the cardiovascular system, skeleton and muscle, gastrointestinal tract, central nervous system, and eyes—and substantial phenotypic overlap across syndromes, coupled with genetic heterogeneity, can complicate diagnosis. Early recognition is therefore important to enable appropriate diagnostic testing and timely, targeted clinical management [1,2].

Noonan syndrome (NS), OMIM #163950, is a condition within the RASopathies spectrum and is characterized by distinctive facial features: high forehead, hypertelorism with down-slanting palpebral fissures, ptosis, a triangular facial shape that becomes more evident with aging, low-set posteriorly rotated ears—typical presentation, but sometimes the craniofacial phenotype may be more subtle—and suggestive presentation [3,4].

Additional features include congenital heart defects, short stature, and variable developmental delay; additional signs may include a broad or webbed neck, chest deformities (superior pectus carinatum and inferior pectus excavatum), coagulation and lymphatic issues, cryptorchidism, hearing loss, and ocular abnormalities. Birth length is usually normal, but adult height typically nears the lower end of normal. Congenital heart diseases occur in about 50–80% of individuals, with pulmonary valve stenosis (PVS) in 20–50% and hypertrophic cardiomyopathy (HCM) in 20–30% (present at birth or developing in infancy or early childhood). Other heart defects can include atrial and ventricular septal defects, branch pulmonary artery stenosis, and tetralogy of Fallot. Intellectual disability is mild, identified in up to 25% of individuals, and language delays are more common in NS than in the general population [5,6,7].

NS is relatively common, with an estimated prevalence of approximately 1 in 1000 to 1 in 2500 individuals. Milder phenotypes may remain unrecognized and therefore be underreported [5]. Diagnosis of NS is established in a proband with suggestive findings and a heterozygous pathogenic variant in *BRAF*, *KRAS*, *MAP2K1*, *MRAS*, *NRAS*, *PTPN11*, *RAF1*, *RASA2*, *RIT1*, *RRAS2*, *SOS1*, or *SOS2* or heterozygous/biallelic pathogenic variants in the *LZTR1* gene, identified by molecular genetic testing. Additional genes associated with Noonan-like phenotypes in a small number of individuals include the *RRAS* and *A2ML1* genes [5,8,9].

**Figure 1:**
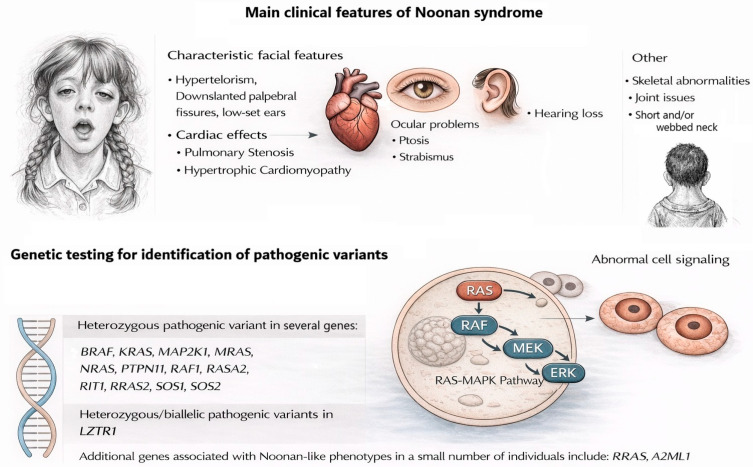
Main clinical features and genetics in Noonan syndrome; diagnosis of Noonan syndrome is established in a proband with suggestive findings and a molecular pathogenic variant identified by genetic testing.

Figure 1 illustrates the primary clinical features and the diagnostic pathway for Noonan syndrome.

Management of NS manifestations generally aligns with standard approaches used in the broader population, particularly for cardiovascular anomalies. Developmental delays are addressed through early intervention programs and individualized educational plans. Clinically significant bleeding is managed according to the underlying coagulation factor deficiencies or platelet dysfunctions. Growth hormone therapy is employed to enhance growth velocity. Conventional treatments are also applied for juvenile myelomonocytic leukemia and other malignancies, feeding difficulties, attention deficit/hyperactivity disorder, behavioral disturbances, male cryptorchidism, renal anomalies (including hydronephrosis), strabismus, hearing impairment, and Chiari malformation [5].

The objective of this study is to describe the clinical and genetic characteristics of Romanian patients diagnosed with NS, mainly using next-generation sequencing. Two cases that were reported before are included in the statistics; the prior paper was published in 2025: Nazarie et al. [10].

## 2. Materials and Methods

### 2.1. Patients

We included 25 patients (17 males, 8 females) evaluated between 2018 and 2025 in Romania at the Emergency Pediatric Clinical Hospital in Cluj-Napoca, the Cluj County Emergency Clinical Hospital, the Clinical Emergency County Hospital in Craiova, and the Heart Institute Cluj-Napoca, all with genetically confirmed diagnoses of NS.

Patients were evaluated clinically, including assessment of family history, auxological data, and dysmorphic features, as well as cardiological evaluation (clinical examination and echocardiography), paraclinical investigations (hematological and coagulation studies, biochemical and hormonal investigations), and additional neurological or psychiatric assessments, when indicated.

In total, 25 patients with genetically confirmed NS were enrolled in this study.

Ethical approval (No. AVZ 148/5 July 2023) for this study was obtained from the Ethics Committee of the “Iuliu Hațieganu” University of Medicine and Pharmacy from Cluj-Napoca, Romania. For patients evaluated prior to the date of ethical approval, clinical and genetic data previously documented in the medical records were retrospectively analyzed, and in some cases, archived biological samples collected during routine clinical care were used for genetic analyses following the granting of ethical approval. A signed written informed consent was obtained from the patients or the legal guardian for all the subjects included in this study.

### 2.2. Genetic Testing

Four patients underwent next-generation sequencing (NGS) with a targeted RASopathy panel in our laboratory. Clinical characteristics of all patients tested through this method are presented in Supplementary Materials Table S1 Supplementary Materials S1.

Additionally, the cohort included two previously published cases diagnosed using PCR followed by restriction enzyme (PCR-RFLP) analysis for *PTPN11* gene [10]. Six patients carrying pathogenic variants were identified by Sanger sequencing of exons 3, 7, and 8 of the *PTPN11* gene. The other 13 patients had been diagnosed externally through targeted gene panels; whole-exome sequencing (WES); or, in one case, whole-genome sequencing (WGS).

For the patients diagnosed in Cluj-Napoca laboratory, peripheral venous blood samples were collected on EDTA for DNA extraction. Genomic DNA was isolated using the Wizard^®^ Genomic DNA Purification Kit (Promega, Madison, WI, USA), following the manufacturer’s protocol. Targeted sequencing was performed using a commercial gene panel (Medicover Genetics TarCET IVD kit, MEDICOVER GENETICS, Nicosia, CYPRUS): familial hypercholesterolemia (FH), pulmonary hypertension (PH) and RASopathies (RAS), covering 30 genes involved in the RAS/MAPK signaling pathway. The panel included the following genes: *AKT3*, *BRAF*, *CBL*, *CCND2*, *EPHB4*, *HRAS*, *KRAS*, *LZTR1*, *MAP2K1*, *MAP2K2*, *MRAS*, *NF1*, *NF2*, *NRAS*, *PIK3CA*, *PIK3R2*, *PPP1CB*, *PTPN11*, *RAF1*, *RASA1*, *RASA2*, *RIT1*, *RRAS*, *SASH1*, *SHOC2*, *SMARCB1*, *SOS1*, *SOS2*, *SPRED1*, and *STAMBP*. Raw data were analyzed using Medicover Genetics’ SIRIUS Data Management Software, and the resulting VCF files were further interpreted on the Geneyx platform [version 6.0, Geneyx Genomex Ltd., Herzliya, Israel], using the human reference genome (hg19/GRCh37).

For the six patients diagnosed by Sanger sequencing, the primer sets used were those described by Tartaglia et al. [11]. Sequencing reactions were prepared with the BigDye™ Terminator v3.1 Cycle Sequencing Kit (Applied Biosystems™, Thermo Fisher Scientific, Waltham, MA, USA), and products were analyzed by capillary electrophoresis on either the 3500 Genetic Analyzer or the SeqStudio Genetic Analyzer, both from Applied Biosystems™. The data were analyzed with the software Unipro Ugene version 53.0 [12].

## 3. Results

Four patients who underwent targeted gene panel sequencing in our laboratory tested positive for pathogenic variants in RASopathy-associated genes. Pathogenic variants were identified in the following genes: *PTPN11*, *SOS1*, and *LZTR1*. Four selected cases with distinctive clinical features are described below.

(a) Patient 1 (see Supplementary Table S1) is a female patient and presented with obstructive cardiomyopathy, large PVS, craniofacial dysmorphism (CFD) characterized by frontal bossing, hypertelorism and low-set ears. The patient also exhibited short stature (below the 3rd percentile). A positive family history was noted, with the father displaying multiple lentigines and wide PVS, and the sister of the patient and paternal grandmother also presented multiple lentigines. Genetic analysis revealed a pathogenic variant in exon 7 of the *PTPN11* gene (p.Tyr279Cys), a variant associated with NSML (**N**oonan **s**yndrome with **m**ultiple **l**entigines, formerly known as Leopard syndrome), confirming the clinical diagnosis.
(b) Patient 2 (see Supplementary Table S1), male, presented with severe PVS, short stature (below the 1st percentile), characteristic facial dysmorphism, a broad thorax, right-sided cryptorchidism, kyphosis with hyperlordosis, and mild intellectual impairment. Genetic testing identified a pathogenic variant in exon 3 of the *PTPN11* gene (p.Ala72Gly).
(c) Patient 3 (see Supplementary Table S1), also a male, presented with PVS, typical facial dysmorphism, and cryptorchidism. A heterozygous pathogenic variant was identified in the *LZTR1* gene (p.Arg284His).
(d) Patient 4 (see Supplementary Table S1) is an adult female who presented with severe PVS, surgically corrected at the age of 33. She exhibited suggestive dysmorphic features (short neck, low-set ears), pectus carinatum, and a stature of 160 cm (32nd percentile). In this case, we identified a pathogenic variant in the *SOS1* gene—the second most frequently implicated gene in NS after *PTPN11*.

Clinical characteristics of all 25 genetically diagnosed patients (including two VUS variants) are presented in Table 1 and a summary of molecular findings is shown in Table 2.

We identified twenty-five positive patients and twenty-six variants, with one patient presenting two pathogenic variants in the *PTPN11* gene, one in exon 3 (c.188A>G) and one in exon 8 (c.922A>G). Unfortunately, parental samples were not available for this case; therefore, we could not determine whether the variants are located in cis or in trans. Twenty-four out of the twenty-six variants (92.31%) were classified as pathogenic and two (7.69%) as variants of uncertain significance (VUS). Regarding sex distribution, males predominated, with a male-to-female ratio of approximately 2:1 (17 males versus 8 females). Two had genetically confirmed inheritance of the pathogenic variant from an affected parent: patient 5 from his mother (patient 6, also described in Table 1), and patient 8 inherited from his father (patient 9, described in Table 1). For four additional patients, one parent was reported to have a suspected clinical diagnosis of NS based on phenotypic features; however, genetic confirmation was not done in these cases. The youngest diagnosed patient (patient 19) was a female infant, two months old, with cardiovascular features (PVS, ostium secundum type atrial septal defect, moderate pulmonary regurgitation, mild tricuspid regurgitation), suggestive CFD (upslanting palpebral fissures, narrow forehead, broad bridge of the nose, thin lips, long philtrum, slightly ogival palate, short neck) and short stature (<p1). The oldest patient diagnosed with NS was also a female (patient 4), 41 years old, presenting severe PVS, suggestive CFD, and pectus carinatum.

The Molecular and ACMG classification of variants identified in patients with Noonan syndrome is detailed in Supplementary Table S2.

**Figure 2:**
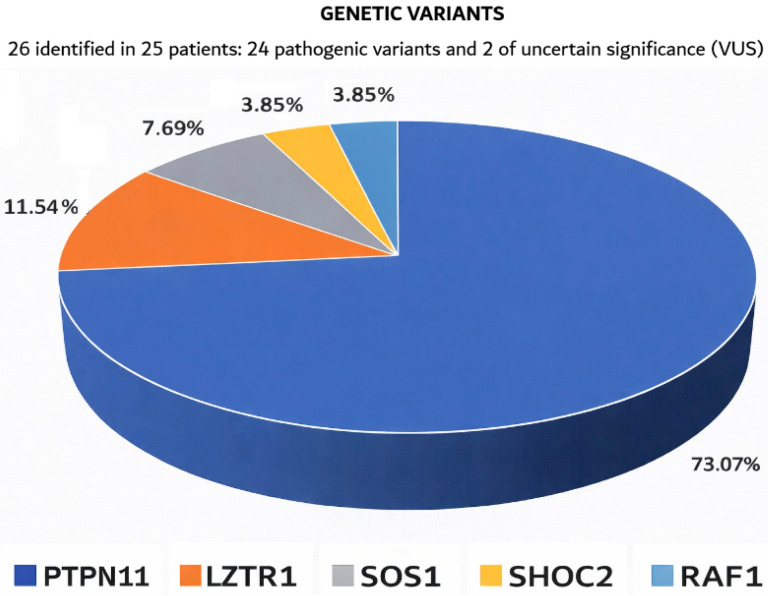
Spectrum of gene variants in RASopathy-associated genes identified in the study cohort (*n* = 25 patients, 26 variants identified).

Distribution of the gene variants in patients studied for NS (including the VUS variants) in the study cohort is shown in Figure 2.

The high frequency of *PTPN11* variants in the total cohort likely reflects ascertainment bias due to targeted hotspot testing in a subset of patients (eight patients tested using Sanger sequencing of specific exons and PCR-RFLP). 

**Figure 3:**
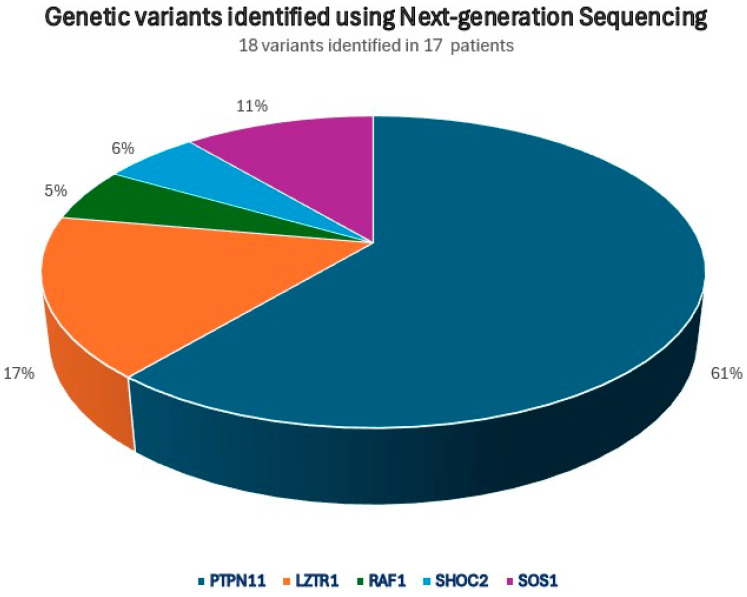
Spectrum of gene variants in RASopathy-associated genes identified in the study cohort using next-generation sequencing (*n* = 17 patients, 18 variants identified).

Figure 3 illustrates the distribution of gene variants, including variants of uncertain significance (VUS), identified via next-generation sequencing in our cohort.

The median age at evaluation for all patients was 8 years, while that of the five patients older than 18 years was 26 years.

Cardiovascular disease was present in 21 out of 25 patients. The remaining four cases included two pediatric patients, who were asymptomatic and did not receive a pediatric cardiology evaluation, and two parents with no prior history of cardiovascular disease, who exhibited clinical features of NS and tested positive for this condition. Although echocardiographic evaluation was recommended, the results were not available at the time of this study. PVS was the most frequent cardiac abnormality, identified in 12 patients (48%). HCM was less common, occurring in only four patients (16%), with one patient presenting both PVS and HCM. Concomitant pulmonary valve stenosis (PVS) and atrial septal defect (ASD) were observed in four patients (16%). Other cardiac findings were: mitral insufficiency (five patients (20%), three of whom also had PVS), PVS and dysplastic pulmonary valve (two patients, 8%), PVS and coarctation of the aorta (one patient, 4%), dysplastic aortic valve (one patient, 4%), mitral regurgitation (three patients, 12%), tricuspid regurgitation (two patients, 8%), aortic regurgitation (one patient, 4%), and pulmonary artery and aortic artery narrowed at the sinotubular junction (STJ) (one patient, 4%). 

Short stature was identified in 16 patients (64% of the cohort). Of these, fourteen patients fell below the 3rd percentile, while two patients were below the 10th percentile. Among the sixteen (64%) patients in our cohort presenting with short stature, only three patients received GH treatment. In seven cases, age was the primary factor: five patients were under the age of 3, and two were diagnosed late, at ages 17 and 27. For the remaining six patients (aged 7–14), GH therapy was not pursued. This was attributed to either a lack of post-diagnostic follow-up or failure to meet the specific national reimbursement criteria in Romania. Romanian therapeutic protocol does not list Noonan syndrome as a primary indication for growth hormone therapy. Consequently, reimbursement is only accessible for NS patients who also meet the diagnostic criteria for either growth hormone deficiency or idiopathic short stature.

CFD, either typical or suggestive, was present in 24 out of the 25 patients. The only patient in whom no dysmorphic features were noted was found to carry a VUS in the *SOS1* gene.

In nine patients (36%), other features were found, including multiple lentigines, ‘café-au-lait’ spots, pigmented nevi, and Sutton nevus, as well as ectodermal findings (sparse or brittle hair, light eyebrows). Five patients (20%) presented dental conditions (multiple cavities, dental dystrophies, malocclusions), while two patients (8%) had eye conditions: one was diagnosed with astigmatism, and the other one with bilateral strabismus. One patient exhibited bilateral hypoacusis requiring hearing aid use from the age of two years; however, this patient was also found to carry a heterozygous pathogenic variant in the *GJB2* gene, suggesting the possibility of an alternative etiology for the hearing impairment. One patient had a clinical history of easy bruising; however, their coagulation parameters were within normal limits. While thrombocyte counts were available for all patients, a full coagulation panel (PT/INR and aPTT) was not systematically performed for the remaining participants in the absence of clinical signs.

All eighteen patients presenting pathogenic variants in the *PTPN11* gene exhibited facial dysmorphism (ten typical, eight suggestive). Additionally, short stature was highly prevalent, affecting sixteen individuals. Co-occurrence of CFD, short stature, and various cardiac conditions was noted in 15 patients. Specifically, a triad of PVS, short stature, and CFD was identified in a subgroup of eight patients, accounting for 50% of those with *PTPN11* pathogenic variants.

We identified two pathogenic variants (patients 10 and 11) affecting the same codon (308) of the *PTPN11* gene, affecting the Y phosphatase domain. In the case of *PTPN11* c.923A>G, the neutral asparagine is substituted by serine (p.Asn308Ser), an amino acid that is also polar and uncharged. In contrast, the other pathogenic variant, *PTPN11* c.922A>G, leads to the replacement of neutral asparagine with negatively charged aspartic acid, which is expected to cause a more pronounced effect.

Three patients were found to harbor pathogenic variants in the *LZTR1* gene; all of them exhibited cardiac involvement (two PVS and one HCM) and typical or suggestive CFD, while none showed short stature.

Variants in *SOS1* were identified in two patients: one classified as pathogenic, and the other as a VUS. Neither patient exhibited stature below the 10th percentile. One patient presented with more clinical features—pulmonary valve stenosis, suggestive dysmorphic features and pectus carinatum—while the other exhibited just multiple lentigines.

Regarding incidental findings, in patients who underwent targeted sequencing (PH, FH, and RAS panels), we identified variants in genes such as *ABCA1*, *ABCG8*, *LPL*, *ACVRL1* and *KCNA5*, but all these variants were classified as VUS based on guidelines of the American College of Medical Genetics and Genomics (ACMG), using ClinVar (https://www.ncbi.nlm.nih.gov/clinvar/, accessed on 13 February 2026), gnomAD v2.1.1 (https://gnomad.broadinstitute.org/, accessed on 13 February 2026). Whole-exome sequencing (WES) performed in patient 25 revealed a pathogenic variant in the gene *ZMYND11* c.744_745del, which could also explain the intellectual disability (ID). RASopathies, such as NS type 5 caused by pathogenic variants in the *RAF1* gene, are commonly associated with mild to moderate developmental delay. Pathogenic variants in *ZMYND11* are also linked to impaired intellectual development with variable severity. Additionally, heterozygous pathogenic variants were identified in two genes, *APOB* and *LDLR* (APOB c.10580G>A, *LDLR* c.1775G>A), both of which are associated with familial hypercholesterolemia.

## 4. Discussion

The multigene sequencing approach highlights the genetic diversity underlying NS in the Romanian cohort, while reinforcing the concept of a unifying pathogenic mechanism at the level of intracellular signal transduction.

As reported in the literature, genes implicated in NS encode components of, or regulators that converge on, the RAS/MAPK cascade, a central signal transduction pathway that integrates extracellular cues to control cell differentiation, metabolism, proliferation, and survival. Upon stimulation by growth factors, cytokines, hormones, and other ligands, adaptor proteins assemble signaling complexes that promote the exchange of GDP for GTP on RAS, converting it from an inactive to an active state. Activated RAS then triggers the RAF–MEK–ERK module through sequential phosphorylation events, leading to ERK activation and nuclear translocation, where it modulates transcriptional programs. Pathogenic variants associated with NS typically increase signaling output through this pathway [5,7].

The genetic landscape of NS, including disease mechanisms and locus information, is summarized in Table 3.

Clinical diagnosis of NS, according to the criteria adapted from Van der Burgt, requires either a typical facial appearance with at least one additional major criterion or two minor criteria or suggestive facial dysmorphology, plus two major or three minor symptoms. The major criteria include short stature (less than percentile 3), chest wall deformities (pectus carinatum/excavatum), cardiac defects (pulmonary valve stenosis, hypertrophic cardiomyopathy, typical ECG), a family history of definite NS, and all of these characteristics: lymphatic dysplasia, ID, and cryptorchidism. Minor criteria comprise other heart problems; a broad thorax; short stature (less than percentile 10); one or more first-degree relatives with suspected NS; and other characteristics such as ID, cryptorchidism, or lymphatic dysplasia [3,24].

Pathogenic variants in the *PTPN11* gene are identified in approximately half of clinically diagnosed NS cases, as previously reported [11,25]. We identified a comparatively high frequency of pathogenic *PTPN11* variants, attributable to targeted diagnostic approaches. Six patients underwent Sanger sequencing, while two were previously diagnosed using PCR with restriction enzyme analysis (PCR-RFLP), representing almost half of our *PTPN11*-positive patients. Although Sanger sequencing remains relatively inexpensive in terms of reagents, it is limited by its low throughput and gene-by-gene approach. This method becomes particularly time-consuming and labor-intensive when multiple exons (e.g., the 16 exons of the *PTPN11* gene) or several candidate genes must be analyzed. In contrast, next-generation sequencing (NGS) enables the simultaneous analysis of multiple genes associated with the same clinical phenotype, making it especially advantageous in genetically heterogeneous disorders. NGS offers higher diagnostic efficiency and broader genomic coverage, but it is associated with higher initial costs and requires more complex bioinformatic analysis [26].

In our cohort, seven patients who fulfilled the Van der Burgt clinical criteria tested positive for pathogenic variants in genes other than *PTPN11*. For these patients, diagnosis through sequential Sanger sequencing would have been challenging, time-consuming, and potentially inconclusive. These findings underscore the clinical utility of NGS-based approaches in disorders with significant genetic heterogeneity. Interestingly, two patients who did not meet the clinical criteria adapted from Van der Burgt [24] were also found to carry relevant variants: one pathogenic variant and one VUS, respectively. One patient, who presented with typical facial dysmorphism, attention deficit and hyperkinetic disorder—thus fulfilling only one major and one minor criterion—had a pathogenic variant in exon 12 of the *PTPN11* gene, c.1403C>T (p.Thr468Met). He was lost to follow-up before a cardiological evaluation could be completed. This variant has been reported in the literature in patients with multiple lentigines [27,28], a clinical feature not observed in our patient. Ahota et al. [28] reported this feature in only one out of six patients who carried this pathogenic variant. The other patient presented with facial features suggestive of a RASopathy, a normal height for his age and gender, but below the target range based on parental heights, multiple lentigines and a normal cardiac evaluation. Given the clinical phenotype, NSML (formerly known as LEOPARD syndrome) or Legius syndrome was clinically suspected, but no pathogenic variant was identified in the *PTPN11*, *RAF1* or *SPRED1* genes. Regarding NSML, according to the recent literature, NSML and NS commonly share pathogenic variants in the *PTPN11* gene [6]. Genetic testing identified a variant, which was classified by the external laboratory as a VUS in the *SOS1* gene. The identified variant (c.3770C>T) is located in the 23rd and final exon of the *SOS1* gene, within the C-terminal domain. It is classified in ClinVar as either likely benign or VUS, with conflicting results from in silico prediction tools. Additionally, the variant has a very low frequency in the general population. Performing familial segregation analysis would provide further insight and could maybe help clarify whether the variant is benign or remains a VUS [29].

The clinical triad comprising PVS, short stature, and craniofacial dysmorphism—identified in 50% of our patients with pathogenic *PTPN11* variants—is consistent with the findings of Shoji et al., who observed this same constellation of symptoms in 58.8% (10/17) of patients harboring *PTPN11* mutations [30].

**Figure 4:**
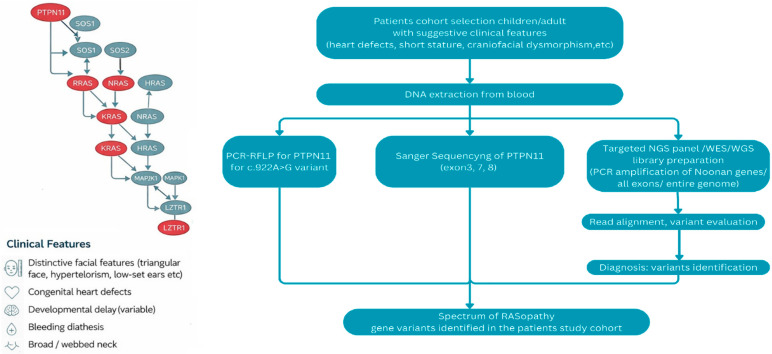
The figure illustrates the integrated clinical and molecular diagnostic strategy used for patients with suspected RASopathies. On the left, the RAS–MAPK signaling pathway is shown, highlighting several genes involved in Noonan spectrum disorders and related conditions (e.g., *PTPN11*, *SOS1*, *KRAS*, *NRAS*, *HRAS*, *MAPK1*, *LZTR1*). Below, the main clinical features prompting genetic testing are summarized, including distinctive craniofacial features, congenital heart defects, etc. On the right, the diagnostic workflow is depicted, starting from patient selection and DNA extraction, followed by Polymerase Chain Reaction–Restriction Fragment Length Polymorphism (PCR-RFLP) for the *PTPN11* c.922A>G variant, Sanger sequencing of selected *PTPN11* exons, parallel molecular approaches including targeted NGS panels, whole-exome sequencing (WES), or whole-genome sequencing (WGS). Sequencing data are processed by read alignment and variant evaluation, leading to molecular diagnosis and definition of the spectrum of RASopathy-associated gene variants identified in the study cohort.

Figure 4 presents the integrated clinical and molecular diagnostic approach applied to the cohort of our patients with suspected RASopathies.

*SOS1* gene variants are associated with NS and hereditary gingival fibromatosis [OMIM entries 610733 and 135300, respectively]. The variant c.1655G>A detected in our patient is reported in cases with multiple giant cell lesions [31,32]. Variations in genes like *SOS1* have been linked to normal stature and a lower incidence of ID or HCM. But sometimes, unusual symptoms such as corpus callosum thickness or left ventricular non-compaction cardiomyopathy (LVNC) can result from some *SOS1* gene mutations [33].

Germline *LZTR1* variants are known to be associated with a broad and variable clinical phenotype, including both NS and schwannomatosis caused by heterozygous loss-of-function variants in *LZTR1* [34]. All reported variants in the *LZTR1* gene were located within the Kelch_4 domain, a critical functional region previously associated with AD pathogenic variants in the *LZTR1* gene [35]. All three variants were reported in patients with schwannomatosis.

A variant affecting the same amino acid residue as the one identified in our study—*LZTR1* c.850C>T (p.Arg284Cys)—has previously been reported in a patient who developed an oligoastrocytoma at 22 years of age, with recurrence as a ganglioglioma at 26 years [34]. Our corresponding variant, *LZTR1* c.851G>A, results in the same amino acid substitution (p.Arg284Cys). Unfortunately, our patient was lost to follow-up, and no additional clinical data were available to assess potential tumor development or long-term outcome. The *LZTR1* c.742G>A variant has previously been reported in individuals with NS who exhibited characteristic clinical features, including craniofacial dysmorphism such as hypertelorism, an ogival palate, and low-set ears, as well as cryptorchidism in males. A range of cardiac anomalies has been described, including PVS, HCM, ASD, and patent ductus arteriosus (PDA), while short stature appears to be less frequent [36,37].

The *LZTR1* c.791+1 G>A variant is predicted to result in aberrant splicing, leading to a frameshift and premature termination codon in exon 9, and has been reported in two siblings with schwannomatoses [34].

Current clinical guidelines indicate that patients with *LZTR1* pathogenic variants may present with pain and have an increased risk of developing unilateral vestibular schwannomas. At diagnosis, patients should undergo high-resolution brain MRI with thin slices (<3 mm) through the internal auditory canals, as well as spinal MRI. If extracranial tumors are detected but remain stable, imaging follow-up is generally recommended every 2–3 years. In contrast, if tumors in the brain are identified, more frequent surveillance (approximately every 12 months) is advised. Imaging frequency should be further increased if new clinical symptoms develop [38]

Pathogenic variants in the *SHOC2* gene are associated with Noonan syndrome-like phenotype with loose anagen hair 1. Patients carrying the same variant have been reported to exhibit features such as skin hyperpigmentation, macrocephaly, loose anagen hair (fine, sparse, or slow-growing), cardiac anomalies, ligamentous laxity, and developmental delay [39]. Our patient, a female, exhibited a combination of clinical signs: PVS, short stature (below the 3rd percentile), suggestive facial dysmorphism, broad nasal base, slight hypertelorism, low-set ears, and a short neck, typical for NS, but also skin hyperpigmentation and mild developmental delay. However, no signs of sparse or slow-growing hair, nor ligamentous laxity, were noted.

The *RAF1* c.1426C>T variant is located within a functional domain and was not found in the gnomAD database. In silico prediction tools (Revel, AlphaMissense) consistently suggest a moderate deleterious effect. This variant lies three nucleotides from a known pathogenic variant (*RAF1* c.1423T>C) previously reported in a patient with NS [40]. However, in the absence of functional studies, this variant is currently classified as a variant of uncertain significance (VUS). While RASopathies are typically associated with mild to moderate developmental delay, the c.1426C>T variant of the *RAF1* gene has not been previously reported in the literature; therefore, we cannot compare our patient’s phenotype with other cases associated with this specific variant. Although in silico prediction suggests a pathogenic effect on the protein, the *RAF1* gene is rarely associated with significant intellectual disability. Consequently, it is more plausible that the cognitive deficiency is linked to the c.744_745del variant of the *ZMYND11* gene, which has an established pathogenic significance for intellectual disability [41]. Furthermore, the left cerebellar atrophy observed in our patient provides additional clinical evidence for associating the *ZMYND11* variant with their neurodevelopmental disorder.

In this patient, the identification of two pathogenic variants in the *APOB* and *LDLR* genes, both associated with FH, likely accounts for the hypercholesterolemia documented in the patient’s medical records. In addition, genetic testing of the patient’s parents and siblings should be considered, particularly for the father, who has suggestive clinical features, including obesity and a myocardial infarction at 42 years of age.

In three patients who underwent targeted RASopathy gene panel sequencing in our laboratory and presented with HCM alongside suggestive facial dysmorphism and short stature, we hypothesized that pathogenic variants in *RAF1*, *NRAS*, or *RIT1*—genes known to be associated with HCM—might be identified in these patients [42]. Additional genes should be analyzed to achieve a definitive molecular diagnosis.

When comparing the prevalence of *RAF1* variants, our cohort included only one patient (4%) harboring a mutation in this gene. In contrast, the study by Ilic et al. [43] on a similarly sized Serbian population (25 patients) identified five patients (20%) with *RAF1* variants. Consequently, the prevalence of HCM was notably lower in our group (4 patients; 16%) compared to the 12 patients (48%) reported in the same study.

Given the inherited nature of these disorders, genetic counseling is an integral part of patient and family care. NS is most commonly inherited in an autosomal dominant (AD) pattern; although many individuals with AD NS carry a *de novo* pathogenic variant, an affected parent is identified in approximately 30–75% of cases. The recurrence risk for siblings depends on parental genetic status: if one parent is affected, the risk is 50%; if both parents are clinically unaffected, the sibling recurrence risk is low (<1%). Each child of an affected individual has a 50% probability of inheriting the pathogenic variant. Pathogenic variants in the *LZTR1* gene may cause NS through either AD or autosomal recessive (AR) inheritance. In AR NS, both parents are typically heterozygous carriers and may be asymptomatic or exhibit mild NS features. Among the 12 families described by Johnston et al., nine couples showed no apparent clinical manifestations. In one family, the maternal grandmother developed unilateral hearing loss in her sixth decade due to a tumor, and several relatives exhibited subtle imaging findings suggestive of schwannomas. In another family, the father presented with mild short stature and ptosis, while in a third family, the mother displayed subtle dysmorphic features, including a mildly broad neck. Of the twelve families, four were consanguineous and had children presenting with clinical features consistent with NS [44]. When both parents are carriers, each pregnancy carries a 25% risk of an affected child, a 50% chance of a heterozygous carrier (potentially with mild features), and a 25% chance of an unaffected, non-carrier child. Prenatal and preimplantation genetic testing can be offered when the causative NS-related pathogenic variant(s) have been identified in the family [5].

## 5. Conclusions

These cases illustrate genotypic heterogeneity in NS. Although the clinical phenotype is almost the same, we found pathogenic variants in different genes. The genetic landscape observed in our cohort is consistent with the established molecular heterogeneity of NS and related RASopathies, with *PTPN11* recognized as the predominant disease-causing gene. Most variants clustered within functionally critical domains, particularly the SH2 and protein tyrosine phosphatase regions, supporting their role in dysregulated RAS–MAPK signaling. Recurrent pathogenic variants further emphasize the presence of mutational hotspots, while the identification of alterations in less frequently implicated genes, such as *LZTR1*, *SOS1*, and *SHOC2*, highlights the importance of comprehensive genetic testing. The detection of VUS also underscores the ongoing challenges in variant interpretation and the need for continued genotype–phenotype correlation studies. Our results highlight the key clinical features that should raise suspicion of NS; genetic confirmation thereby enables appropriate multidisciplinary management involving cardiologists, endocrinologists, neurologists, pediatricians, geneticists, and other specialists. Though based on a modest-sized cohort, our study adds phenotypic and genetic insights into Noonan syndrome within the Romanian population.

## Figures and Tables

**Table 1:** Synthesis of the clinical features of the 25 patients with a molecular diagnosis, including two carrying variants of uncertain significance (VUS).

P	Sex	Age at Evaluation	PVS (Severity)	HCM (Severity)	Other Cardiac Features	Typical CFD	Suggestive CFD	Height <p3	Height <p10	Pectus Carinatum/Excavatum	Broad Thorax	1st Degree Relative with Definite Signs of NS	1st Degree Relative with Suggestive Signs of NS #	Family History	CO	ID	Other Features	Growth Hormone Therapy	Affected Gene
1	F	7	+ (mild)	+ (mild)	Mitral insufficiency grade I/II.	−	+	+	na	−	−	−	+	Father: multiple lentigines; sister and paternal grandmother: lentigines, mild PS	na	−	Multiple lentigines. Multiple teeth cavities	No	*PTPN11*
2	M	10	+ (severe)	−	Severe supravalvular PVS; ASD, type: wide OS. Pulmonary insufficiency grade II. Mitral insufficiency grade II.	+	−	+	na	−	+	−	−	ns	+	+	Dental dystrophies. Ligament hyperlaxity. Dorsal kyphoscoliosis, lumbar hyperlordosis	No	*PTPN11*
3	M	2	+	−	−	+		−	−	−	−	−	−	ns	+	−	−	No	*LZTR1*
4	F	41	+ (severe)	−	ASD, type: OS, closed by patch at 33 years. Mitral insufficiency grade II. Tricuspid insufficiency grade I/II.	−	+	−	−	+ (carinatum)	−	−	−	ns	na	−	−	No	*SOS1*
5	M	2	+ (moderate)	−	Moderate PVS, moderate supra-valvular stenosis. Dysplastic pulmonary valve.	+	−	+	na	−	−	−	+	Mother with short stature and craniofacial dysmorphism	+	−	−	No	*PTPN11*
6	F	26	ne	ne	ne	−	+	ne	ne	−	−	+	−	A son diagnosed with NS ^1^	na	−	−	No	*PTPN11*
7	M	17	+	−	−	−	+	+	na	−	−	−	−	ns	+	−	Pigmented nevi on the neck, face, and chest. Dental implantation abnormalities, and dental malocclusion	No	*PTPN11*
8	M	5	−	−	Minor mitral insufficiency. ASD, type: small OS, with left–right shunt.	+	−	+	na	+ (excavatum)	−	+	−	Father with typical facial dysmorphism, short stature: 158 cm (<percentile 1)	−	+	Hypoplastic teeth	Yes	*PTPN11*
9	M	27	ne	ne	ne	+	−	+	na	−	−	+	−	A son diagnosed with NS ^2^	−	−	−	No	*PTPN11*
10	M	14	−	−	Pulmonary valve dysplasia. Pulmonary artery ectasia.	−	+	+	na	−	−	−	−	ns	+	−	Easy bruising. Surgery for sensory hearing loss ^3^	No	*PTPN11*
11	M	1	+ (severe)	−	Severe supravalvular pulmonary stenosis.	+	−	+	na	−	−	−	−	ns	−	ne ^4^	−	No	*PTPN11*
12	M	8	−	−	Minor aortic insufficiency and minor mitral insufficiency.	−	+	+	na	+ (carinatum)	−	−	−	ns	−	−	−	Yes	*PTPN11*
13	F	8	+ (severe)	−	−	−	+	+	na	−	−	−	−	ns	na	−	−	No	*PTPN11*
14	M	8	−	−	Mild aortic regurgitation, dysplastic aortic valve.	−	+	+	na	+ (excavatum)	−	−	+	Mother under observation with NS specific phenotype	−	−	Pigmented nevi on posterior thorax: “café-au-lait” spots on left arm and anterior left thigh	No	*PTPN11*
15	M	2	ne	ne	ne	+	−	−	−	−	−	−	−	ns	−	+	−	No	*PTPN11*
16	M	10	−	−	Pulmonary artery narrowed at the STJ. Ascending aorta slightly narrowed at the STJ.	−	+	−	−	−	−	−	−	ns	−	−	Astigmatism	No	*PTPN11*
17	M	12	+ (mild)	−	Aortic coarctation. Mild pulmonary and tricuspid regurgitation. Small ASD with left–right shunt. Thin interatrial sept.	+	−	+	na	+ (excavatum)	−	−	+	Sister: deceased hydrocephalus; mother presents NS-specific phenotype	+	−	Multiple pigmented nevi at scapular level	Yes	*PTPN11*
18	M	1	−	+	−	−	+	−	+	+ (carinatum)	−	−	−	ns	−	−	Multiple small “café-au-lait” spots on the posterior chest, scalp, axillae, sparse and light eyebrows, brittle and thin hair	No	*PTPN11*
19	F	0	+	−	ASD, type OS. Moderate pulmonary regurgitation. Mild tricuspid regurgitation.	−	+	−	−	+ (excavatum)	−	−	−	ns	na	ne ^4^	−	No	*PTPN11*
20	F	0	−	+ (severe)	Severe mitral regurgitation and 1st-degree atrio-ventricular block.	−	+	+	na	−	−	−	−	ns	na	ne ^4^	−	No	*PTPN11*
21	F	13	+	−	Residual pulmonary insufficiency; mitral valve prolapse; 1st-degree mitral insufficiency; asymptomatic ventricular extra-systolic arrhythmia with RBBB morphology.		+	+	na	−	−	−	−	ns	na	+	Teeth implantation defects	No	*SHOC2*
22	F	19	−	+	Mild mitral regurgitation and infundibular stenosis of the RV le infundibular stenosis.		+	−	−	−	−	−	−	ns	na	−	−	No	*LZTR1*
23	M	15	+ (mild)	−	Dysplastic pulmonary valve RV hypertrophy.	+	−	−	−	−	−	−	−	ns	+	−	Multiple pigmented nevi, Sutton nevus	No	*LZTR1*
24	M	5	ne	ne	ne ^5^	−	−	−	−	−	−	−	−	ns	−	−	Multiple lentigines	No	*SOS1*
25	M	25	−	−	Mild mitral regurgitation and concentric LV remodeling.	−	+	−	−	−	−	−	−	Father: grade 2 obesity; down-slanting palpebral fissures; bulbous nose; myocardial infarction at age 42	−	+	−	No	*RAF1* ^6^

Legend: AO—aortic coarctation; ASD—atrial septal defect; CFD—craniofacial dysmorphism; CO—cryptorchidism; HCM—hypertrophic cardiomyopathy; ID—intellectual disability; LV—left ventricle; NS—Noonan syndrome; P—patient; PVS—pulmonary valve stenosis; RBBB—right bundle branch block; OS—ostium secundum; RV—right ventricle; STJ—sinotubular junction; na—not applicable; ns—not-significant; ne—not evaluated. + means present; − means not present. ^1^ The mother of patient 5. ^2^ The father of patient 8. ^3^ The patient is also heterozygous for *GJB2* 35delG. ^4^ The children are 1 year old or under 1 year old; intellectual disability was not evaluated. ^5^ The patient was lost to follow-up after genetic testing and did not undergo echocardiographic assessment. The father’s height was 185 cm, and the mother’s height was 162 cm. Although the child’s height falls within the 64th percentile compared to peers of the same age, it remains below the expected target height range based on parental stature. ^6^ The patient also carries pathogenic variants in the following genes: *ZMYND11* c.744_745del, *APOB* c.10580G>A, and *LDLR* c.1775G>A. # Only two cases have genetically confirmed inheritance, while several additional cases may represent suspected familial transmission based on clinical features in a parent.

**Table 2:** Summary of molecular findings in the study cohort, including the patient identifier, affected gene, variant description, variant type, ACMG classification, exon location, gene domain, and diagnostic method.

Patient	Gene	Variant	Type	ACMG Classification	Exon	Gene Domain	Method
1	*PTPN11*	c.836A>G (p.(Tyr279Cys))	Missense	P	7	Y phosphatase	Targeted gene panel sequencing
2	*PTPN11*	c.215C>G (p.(Ala72Gly))	Missense	P	3	N-SH2	Targeted gene panel sequencing
3	*LZTR1*	c.851G>A (p.(Arg284His))	Missense	P	9	Kelch repeat domain	Targeted gene panel sequencing
4	*SOS1*	c.1655G>A (p.(Arg552Lys))	Missense	P	10	-	Targeted gene panel sequencing
5	*PTPN11*	c.236A>G (p.(Gln79Arg))	Missense	P	3	N-SH2	Sanger sequencing
6	*PTPN11*	c.236A>G (p.(Gln79Arg))	Missense	P	3	N-SH2	Sanger sequencing
7	*PTPN11*	c.214G>T (p.(Ala72Ser))	Missense	P	3	N-SH2	Sanger sequencing
8	*PTPN11*	c.844A>G (p.(Ile282Val))	Missense	P	7	Y phosphatase	Sanger sequencing
9	*PTPN11*	c.844A>G (p.(Ile282Val))	Missense	P	7	Y phosphatase	Sanger sequencing
10	*PTPN11*	c.923A>G (p.(Asn308Ser))	Missense	P	8	Y phosphatase	Sanger sequencing
11	*PTPN11*	c.922A>G (p.(Asn308Asp))	Missense	P	8	Y phosphatase	PCR-RFLP
12	*PTPN11*	c.922A>G (p.(Asn308Asp))	Missense	P	8	Y phosphatase	PCR-RFLP
13	*PTPN11*	c.179G>C (p.(Gly60Ala))	Missense	P	3	N-SH2	WGS
14	*PTPN11*	c.188A>G (p.(Tyr63Cys))/ c.922A>G (p.(Asn308Asp))	Missense	P	3/8	N-SH2/ Y phosphatase	Targeted gene panel sequencing
15	*PTPN11*	c.1403C>T (p.(Thr468Met))	Missense	P	12	Y phosphatase, DSPc	Targeted gene panel sequencing
16	*PTPN11*	c.1522A>G (p.(Met508Val))	Missense	P	13	Y phosphatase, DSPc	WES
17	*PTPN11*	c.1471C>T (p.(Pro491Ser))	Missense	P	13	Y phosphatase, DSPc	Targeted gene panel sequencing
18	*PTPN11*	c.1528C>G (p.(Gln510Glu))	Missense	P	13	Y phosphatase, DSPc	Targeted gene panel sequencing
19	*PTPN11*	c.1504T>G (p.(Ser502Ala))	Missense	P	Exon 13	Y phosphatase, DSPc	Targeted gene panel sequencing
20	*PTPN11*	c.1528C>G (p.(Gln510Glu))	Missense	P	13	Y phosphatase, DSPc	Targeted gene panel sequencing
21	*SHOC2*	c.4A>G (p.(Ser2Gly))	Missense	P	2	-	Targeted gene panel sequencing
22	*LZTR1*	c.791+1 G>A (p.?)	Splice donor	P	8	Kelch repeat domain	Targeted gene panel sequencing
23	*LZTR1*	c.742G>A (p.(Gly248Arg))	Missense	P	8	Kelch repeat domain	WES
24	*SOS1*	c.3770C>T (p.(Thr1257Ile))	Missense	VUS	23	-	Targeted gene panel sequencing
25	*RAF1*	c.1426C>T (p.(Leu476Phe))	Missense	VUS	14	Protein kinase domain	WES

Legend: ACMG—American College of Medical Genetics and Genomics; DSPc—Dual-Specificity Phosphatase, catalytic domain; P—pathogenic; VUS—variant of uncertain significance. Variants are reported according to HGVS nomenclature using the following reference transcripts: *PTPN11* (NM_002834.5, NM_001330437.2 for patient 16), *SOS1* (NM_005633.4), *LZTR1* (NM_006767.4), *SHOC2* (NM_007373.3), and *RAF1* (NM_002880.3). WES—whole-exome sequencing; WGS—whole-genome sequencing; Y phosphatase—tyrosine protein phosphatase domain.

**Table 3:** Genes implicated in NS—underlying disease mechanisms, relative contribution, and compiled data useful for molecular genotyping—from the standard references: gene HGMD (Human Gene Mutation Database), chromosome locus from OMIM (Online Mendelian Inheritance in Man), and protein from UniProt.

Gene	Chromosome Locus	Protein	Mechanism	Proportion of NS Cases Attributed to Pathogenic Variants in Each Gene (%)	References	Variants in Present Study (Pathogenic + VUS)
*BRAF*	7q34	Serine/threonine protein kinase B-raf	Gain of function	<2%	[13]	0
*KRAS*	12p12.1	GTPase KRas	Gain of function	<5%	[5]	0
*LZTR1*	22q11.21	Leucine zipper-like transcriptional regulator 1	Gain of function	~8%	For heterozygous pathogenic variants that lead to AD LZTR1-related NS [14]	3
	22q11.21	Leucine zipper-like transcriptional regulator 1	Loss of function		Recessive variants (AR) typically influence protein synthesis/stability or subcellular localization [14]	0
*MAP2K1*	15q22.31	Dual-specificity mitogen-activated protein kinase 1	Gain of function	<2%	[15,16]	0
*MRAS*	3q22.3	Ras-related protein M-Ras		<1%	[14,17]	0
*NRAS*	1p13.2	GTPase NRas		<1%	[5]	0
*PTPN11*	12q24.13	Tyrosine protein phosphatase non-receptor type 11		50%	[5]	18
*RAF1*	3p25.2	RAF proto-oncogene serine/threonine protein kinase		5%	[18]	1 (VUS)
*RASA2*	3q23	Ras GTPase-activating protein 2		Unknown	[19]	0
*RIT1*	1q22	GTP-binding protein Rit1		5%	[20]	0
*RRAS2*	11p15.2	Ras-related protein R-Ras2		<1%	[8,19]	0
*SOS1*	2p22.1	Son of sevenless homolog 1		10–13%	[21]	1 (+1 VUS)
*SOS2*	14q21.3	Son of sevenless homolog 2		~4%	[19,22,23]	0
Others, Noonan syndrome-like phenotype SHOC2	10q25.2	Leucine-rich repeat protein SHOC-2		2%	[5]	1

Legend: AD—autosomal dominant; AR—autosomal recessive; VUS—variant of uncertain significance.

## Data Availability

The original contributions presented in this study are included in the article/Supplementary Material. Further inquiries can be directed to the corresponding author.

## Supplementary Materials

The following supporting information can be downloaded at https://www.mdpi.com/article/10.3390/diagnostics16081207/s1. Table S1: Clinical characterization and secondary findings in the first group: 13 patients who underwent targeted RASopathy gene panel sequencing using Medicover Genetics TarCET IVD kit for familial hypercholesterolemia (FH), pulmonary hypertension (PH), and a RASopathy panel covering 30 genes involved in the RAS/MAPK signaling pathway. Table S2. Molecular and ACMG classification of variants identified in patients with Noonan syndrome.

## Abbreviations

The following abbreviations are used in this manuscript:

ACMG	American College of Medical Genetics and Genomics
AD	Autosomal dominant
AR	Autosomal recessive
ASD	Atrial septal defect
CO	Cryptorchidism
CFD	Craniofacial dysmorphism
DSPc	Dual-Specificity Phosphatase, catalytic domain
FH	Familial hypercholesterolemia
GDP	Guanosine diphosphate
GTP	Guanosine triphosphate
HCM	Hypertrophic cardiomyopathy
HGMD	Human Gene Mutation Database
HGVS	Human Genome Variation Society
ID	Intellectual disability
LVNC	Left ventricular non-compaction cardiomyopathy
MAPK	Mitogen-activated protein kinase pathway
NGS	Next-generation sequencing
NS	Noonan syndrome
NSML	Noonan syndrome with multiple lentigines, formerly known as LEOPARD syndrome
OMIM	Online Mendelian Inheritance in Man
OS	Ostium secundum
PCR-RFLP	Polymerase Chain Reaction–Restriction Fragment Length Polymorphism
PDA	Patent ductus arteriosus
PH	Pulmonary hypertension
PTPN11	Protein Tyrosine Phosphatase, Non-Receptor Type 11
PVS	Pulmonary valve stenosis
RAS/MAPK	Rat Sarcoma-/Mitogen-Activated Protein Kinase
RBBB	Right bundle branch block
STJ	Sinotubular junction
VUS	Variant of uncertain significance
