# Supplementary material for: Molecular Diagnosis and Phenotypic Variability of Noonan Syndrome: Experience from a Romanian Multicenter Study
Source: Diagnostics (Basel). 2026 Apr 17;16(8):1207. doi: 10.3390/diagnostics16081207 (PMC13114569; doi:10.3390/diagnostics16081207)
Supplement: Supplementary file 1 [file diagnostics-16-01207-s001.zip › diagnostics-4199055-supplementary.pdf]

Table S1. Clinical characterization and secondary findings in the first group: 13 patients who underwent targeted RASopathy gene panel sequencing using Medicover Genetics TarCET IVD kit for familial hypercholesterolemia (FH), pulmonary hypertension (PH), and a RASopathy panel covering 30 genes involved in the RAS/MAPK signaling pathway.

Patient	Sex	Age at evaluation	PVS	HCM	Other cardiac signs	Typical craniofacial dysmorphism	Suggestive craniofacial dysmorphism	Height <p3	Height <p10	Pectus carinatum/pectus excavatum	Broad Thorax	First-degree relative with definite NS	First degree with suggestive	CO	MR	Other features	Gene	Variant	Type	Secondary findings
1	F	7 years	+	+	-	-	+	+	na	-	-	-	+	-	-	Multiple lentigines	PTPN11	c.836A>G<br>p.Tyr279Cys<br>heterozygous	P	-
2	M	10 years	+	-	-	+	-	+	na	-	+	-	-	+	+	Dental dystrophies, ligament hyperlaxity, dorsal kyphoscoliosis, lumbar hyperlordosis	PTPN11	c.215C>G<br>p.Ala72Gly<br>heterozygous	P	*ABCA1* : c.6591C>A<br>p.Ser2197Arg<br>heterozygous
3	M	2 years	+	-	-	+	-	-	-	-	-	-	-	+	-	-	LZTR1	c.851G>A<br>p.Arg284His<br>heterozygous	P	-
4	F	41 years	+	-	-	-	+	-	-	+/<br>carinatum	-	-	-	-	-	-	Sos1	c.1655G>A<br>p.Arg552Lys<br>heterozygous	P	-
5	M	2 months	-	+	-	+	-	-	-	-	-	-	-	-	-	-	SOS1[1]	c.836T>A<br>p.Val279Glu<br>homozygous	VUS	-
6	M	3 years	+	-	-	-	+	-	-	-	-	-	-	+	-	-	Negative	-		*ABCA1*:c.6590del<br>p.Ser2197Thrfs*7<br>VUS<br>heterozygous
7	F	3 years	+	-	-	+	-	-	-	-	-	-	-	-	-	-	Negative	-	-	*ABCA1*:c.6596_6597insC<br>p.Lys2199Asnfs*16<br>VUS<br>heterozygous
8	F	66 years	+	-	-	-	+	-	+	-	-	-	-	-	-	-	Negative	-	-	KCNA5:c.497A>C<br>p.Asp166Ala<br>VUS[2]<br>Heterozygous<br><br>*ABCA1*:c.6590del<br>p.Ser2197Thrfs*7<br>VUS<br>Heterozygous;<br><br>*ABCA1*:c.6596_6597insC<br>p.Lys2199Asnfs*16<br>VUS<br>Heterozygous
9	M	52 years	-	+	-	-	+	+	na	-	-	-	-	-	-	-	Negative	-	-	-
10	F	2 years	+	-	-	-	+	-	+	-	-	-	-	-	-	-	Negative	-	-	ABCA1:c.6591C>A<br>p.(Ser2197Arg)<br>VUS<br>heterozygous
11	F	42 years	-	+	-	-	+	-	-	-	-	-	-	-	-	-	Negative	-	-	-
12	F	1 year	+	-	-	-	+	+	na	-	-	-	-	-	-	-	Negative	-	-	-

Patient	Sex	Age at evaluation	PVS	HCM	Other cardiac signs	Typical craniofacial dysmorphism	Suggestive craniofacial dysmorphism	Height <p3	Height <p10	Pectus carinatum/pectus excavatum	Broad Thorax	First-degree relative with definite NS	First degree with suggestive	CO	MR	Other features	Gene	Variant	Type	Secondary findings
13	F	1 year	-	-	+	-	+	+	na	-	-	-	-	-	-	-	Negative	-	-	*ABCA1*:c.6596_6597insC p.Lys2199Asnfs*16 VUS Heterozygous; *ABCA1*:c.6590del p.Ser2197Thrfs*7 VUS Heterozygous;

1. Sanger sequencing was performed to validate the variant; however, it could not be confirmed and therefore was considered a likely technical artifact. Consequently, it was excluded from all final results.
2. Pathogenic variants in the KCNA5 gene are associated with atrial fibrillation (MIM 612240). In addition, variants affecting the BTB_2 domain have been linked to loss of potassium channel function and may contribute to the development of pulmonary arterial hypertension.

CO- Cryptorchidism; P- pathogenic, PVS – Pulmonary Valve Stenoses, HCM- Hypertrophic cardiomyopathy, MR- Mental retardation, + present, - not present, na- not applicable

Table S2. Molecular and ACMG classification of variants identified in patients with Noonan syndrome.

Patient	Gene	Variant	Type	ACMG Classification	ACMG Criteria	Exon	Gene domain	Method
1	*PTPN11*	c.836A>G (p.(Tyr279Cys))	Missense	P	PP1, PM2, PM5, PM1, PP3, PP2, PS3, PP5	7	Y phosphatase	Targeted gene panel sequencing
2	*PTPN11*	c.215C>G (p.(Ala72Gly))	Missense	P	PM2, PM5, PM1, PP3, PP2, PS3, PP5	3	N-SH2	Targeted gene panel sequencing
3	*LZTR1*	c.851G>A (p.(Arg284His))	Missense	P	PS4, PM2, PM5, PP3, PM1, PP5	9	Kelch repeat domain	Targeted gene panel sequencing
4	*SOS1*	c.1655G>A (p.(Arg552Lys))	Missense	P	PM1, PS3, PM2, PM5, PS4, PP3, PP5	10	-	Targeted gene panel sequencing
5	*PTPN11*	c.236A>G (p.(Gln79Arg))	Missense	P	PP1, PM2, PM5, PM1, PP3, PP2, PS3, PP5	3	N-SH2	Sanger sequencing
6	*PTPN11*	c.236A>G (p.(Gln79Arg))	Missense	P	PP1, PM2, PM5, PM1, PP3, PP2, PS3, PP5	3	N-SH2	Sanger sequencing
7	*PTPN11*	c.214G>T (p.(Ala72Ser))	Missense	P	PP1, PM2, PM5, PM1, PP3, PP2, PS3, PP5	3	N-SH2	Sanger sequencing
8	*PTPN11*	c.844A>G (p.(Ile282Val))	Missense	P	PS4, PM2, PM5, PM1, PP2, PS3, PP1, PP5	7	Y phosphatase	Sanger sequencing
9	*PTPN11*	c.844A>G (p.(Ile282Val))	Missense	P	PS4, PM2, PM5, PM1, PP2, PS3, PP1, PP5	7	Y phosphatase	Sanger sequencing
10	*PTPN11*	c.923A>G (p.(Asn308Ser))	Missense	P	PP1, PM2, PM5, PP3, PM1, PP2, PS3, PP5	8	Y phosphatase	Sanger sequencing
11	*PTPN11*	c.922A>G (p.(Asn308Asp))	Missense	P	PS3, PP1, PM2, PM5, PP3, PM1, PP2, PP5	8	Y phosphatase	PCR-RFLP
12	*PTPN11*	c.922A>G (p.(Asn308Asp))	Missense	P	PS3, PP1, PM2, PM5, PP3, PM1, PP2, PP5	8	Y phosphatase	PCR-RFLP
13	*PTPN11*	c.179G>C (p.(Gly60Ala))	Missense	P	PP3, PM2, PM5, PP2, PS3, PP5	3	N-SH2	WGS
14	*PTPN11*	c.188A>G (p.(Tyr63Cys)) / c.922A>G (p.(Asn308Asp))	Missense	P	PS1,PS3,PP1,PM2,PM1,PS4,PP3,PP2,PP5 / PS3, PP1, PM2, PM5, PP3, PM1, PP2, PP5	3 / 8	N-SH2/ Y phosphatase	Targeted gene panel sequencing
15	*PTPN11*	c.1403C>T (p.(Thr468Met))	Missense	P	PS3, PP1, PM2, PM5, PS4, PP3, PM1, PP2, PP5	12	Y phosphatase, DSPc	Targeted gene panel sequencing
16	*PTPN11*	c.1522A>G (p.(Met508Val))	Missense	P	PS3, PM2, PM1, PS4, PP3, PP2, PP5	13	Y phosphatase, DSPc	WES
17	*PTPN11*	c.1471C>T (p.(Pro491Ser))	Missense	P	PS4, PM2, PM5, PM1, PP2, PS3, PP5	13	Y phosphatase, DSPc	Targeted gene panel sequencing
18	*PTPN11*	c.1528C>G (p.(Gln510Glu))	Missense	P	PM2, PM5, PM1, PP3, PP2, PS3, PP5	13	Y phosphatase, DSPc	Targeted gene panel sequencing
19	*PTPN11*	c.1504T>G (p.(Ser502Ala))	Missense	P	PM2, PM5, PM1, PP3, PP2, PP5	Exon 13	Y phosphatase, DSPc	Targeted gene panel sequencing
20	*PTPN11*	c.1528C>G (p.(Gln510Glu))	Missense	P	PM2, PM5, PM1, PP3, PP2, PS3, PP5	13	Y phosphatase, DSPc	Targeted gene panel sequencing
21	*SHOC2*	c.4A>G (p.(Ser2Gly))	Missense	P	PS3, PM2, PS4, PP5	2	-	Targeted gene panel sequencing
22	*LZTR1*	c.791+1 G>A (p.?)	Splice donor	P	PVS1, PM2, PS4, PP5	8	Kelch repeat domain	Targeted gene panel sequencing
23	*LZTR1*	c.742G>A (p.(Gly248Arg))	Missense	P	PS3, PM2, PM5, PM1, PP3, PP5	8	Kelch repeat domain	WES

24	*SOS1*	c.3770C>T (p.(Thr1257Ile))	Missense	VUS	PM2, BP6	23	-	Targeted gene panel sequencing
25	*RAF1*	c.1426C>T (p.(Leu476Phe))	Missense	VUS	PM2, PP3	14	Protein kinase domain	WES

Legend: P- Pathogenic; VUS- Variant of uncertain significance; variants are reported according to HGVS nomenclature using the following reference transcripts: *PTPN11* (NM_002834.5, NM_001330437.2 for patient 16), *SOS1* (NM_005633.4), *LZTR1* (NM_006767.4), *SHOC2* (NM_007373.3), *RAF1* (NM_002880.3);

ACMG criteria published by Richards et all [1]

PVS1 - null variant in a gene where loss of function is a known mechanism of disease

PS3 - well-established functional studies show damaging effect on the gene or gene product

PS4 - the prevalence of the variant in affected individuals is significantly higher than its prevalence in unaffected controls
PP1 - co-segregation with disease in multiple affected family members in a gene definitively known to cause the disease
PP2 - missense variant in a gene that has a low rate of benign missense variation and where missense variants are a common mechanism of disease
PP3 - for a missense or a splicing region variant, computational prediction tools unanimously support a deleterious effect on the gene
PP5 - reputable source recently reports variant as pathogenic, but the evidence is not available to the laboratory to perform an independent evaluation
PM1- non-truncating non-synonymous variant is located in a mutational hot spot and/or critical and well-established functional domain
PM2 - extremely low frequency in gnomAD population databases
PM5 - different amino acid change as a known pathogenic varian
PP5 - reputable source recently reports variant as pathogenic but the evidence is not available to the laboratory to perform an independent evaluation
BP6 - reputable source recently reports variant as benign but the evidence is not available to the laboratory to perform an independent evaluation

Reference

[1] Richards, S.; Aziz, N.; Bale, S.; Bick, D.; Das, S.; Gastier-Foster, J.; Grody, W. W.; Hegde, M.; Lyon, E.; Spector, E.; Voelkerding, K.; Rehm, H. L. Standards and Guidelines for the Interpretation of Sequence Variants: A Joint Consensus Recommendation of the American College of Medical Genetics and Genomics and the Association for Molecular Pathology. *Genet. Med.* **2015**, *17* (5), 405–424. https://doi.org/10.1038/gim.2015.30.
